# Connectivity derived thalamic segmentation in deep brain stimulation for tremor

**DOI:** 10.1016/j.nicl.2018.01.008

**Published:** 2018-01-28

**Authors:** Harith Akram, Viswas Dayal, Philipp Mahlknecht, Dejan Georgiev, Jonathan Hyam, Thomas Foltynie, Patricia Limousin, Enrico De Vita, Marjan Jahanshahi, John Ashburner, Tim Behrens, Marwan Hariz, Ludvic Zrinzo

**Affiliations:** a Unit of Functional Neurosurgery, Sobell Department of Motor Neuroscience and Movement Disorders, UCL Institute of Neurology, Queen Square, London WC1N 3BG, UK; b Victor Horsley Department of Neurosurgery, National Hospital for Neurology and Neurosurgery, Queen Square, London WC1N 3BG, UK; c Department of Clinical Neuroscience, Umeå University, Umeå, Sweden; d Wellcome Trust Centre for Neuroimaging, UCL Institute of Neurology, Queen Square, London WC1N 3BG, UK; e Centre for Functional MRI of the Brain (FMRIB), John Radcliffe Hospital, Oxford OX3 9DU, UK; f Neuroradiological Academic Unit, Department of Brain Repair and Rehabilitation, UCL Institute of Neurology, Queen Square, London WC1N 3BG, UK; g Lysholm Department of Neuroradiology, National Hospital for Neurology and Neurosurgery, University College London NHS Foundation Trust, London, UK; h Department of Neurology, Innsbruck Medical University, Innsbruck, Austria

**Keywords:** AC, anterior commissure, BEDPOSTX, Bayesian estimation of diffusion parameters obtained using sampling techniques X, BET, brain extraction tool, CI, confidence interval, CON, connectivity, DBS, deep brain stimulation, DF, degrees of freedom, DICOM, digital imaging and communications in medicine, DWI, diffusion weighted imaging, EV, explanatory variable, FLIRT, FMRIB's linear image registration tool, FMRIB, Oxford centre for functional MRI of the brain, FNIRT, FMRIB's non-linear image registration tool, FoV, field of view, FSL, FMRIB's software library, GLM, general linear model, HARDI, high angular resolution diffusion imaging, HFS, high frequency stimulation, IPG, implantable pulse generator, LC, Levodopa challenge, LEDD, l-DOPA equivalent daily dose, M1, primary motor cortex, MMS, mini-mental score, MNI, Montreal neurological institute, MPRAGE, magnetization-prepared rapid gradient-echo, MPTP, 1-methyl-4-phenyl-1,2,3,6-tetrahydropyridine, NHNN, National Hospital for Neurology and Neurosurgery, NIfTI, neuroimaging informatics technology initiative, PC, posterior commissure, PFC, prefrontal cortex, PMC, premotor cortex, S1, primary sensory cortex, SAR, specific absorption rate, SD, standard deviation, SE, standard error, SMA, supplementary motor area, SNR, signal-to-noise ratio, SSEPI, single-shot echo planar imaging, STN, subthalamic nucleus, TFCE, threshold-free cluster enhancement, TMS, transcranial magnetic stimulation, UPDRS, unified Parkinson's disease rating scale, VBM, voxel based morphometry, VL, ventral lateral, VP, ventral posterior, VTA, volume of tissue activated, cZI, caudal zona incerta, Diffusion weighted imaging, DWI, Connectivity, Parkinson's disease, PD, Ventrointermedialis, VIM, Dentato-rubro-thalamic tract, DRT, Ventrolateral nucleus, VL, Dentate nucleus, Tremor, Deep brain stimulation, DBS

## Abstract

The ventral intermediate nucleus (VIM) of the thalamus is an established surgical target for stereotactic ablation and deep brain stimulation (DBS) in the treatment of tremor in Parkinson's disease (PD) and essential tremor (ET). It is centrally placed on a cerebello-thalamo-cortical network connecting the primary motor cortex, to the dentate nucleus of the contralateral cerebellum through the dentato-rubro-thalamic tract (DRT). The VIM is not readily visible on conventional MR imaging, so identifying the surgical target traditionally involved indirect targeting that relies on atlas-defined coordinates. Unfortunately, this approach does not fully account for individual variability and requires surgery to be performed with the patient awake to allow for intraoperative targeting confirmation. The aim of this study is to identify the VIM and the DRT using probabilistic tractography in patients that will undergo thalamic DBS for tremor. Four male patients with tremor dominant PD and five patients (three female) with ET underwent high angular resolution diffusion imaging (HARDI) (128 diffusion directions, 1.5 mm isotropic voxels and b value = 1500) preoperatively. Patients received VIM-DBS using an MR image guided and MR image verified approach with indirect targeting. Postoperatively, using parallel Graphical Processing Unit (GPU) processing, thalamic areas with the highest diffusion connectivity to the primary motor area (M1), supplementary motor area (SMA), primary sensory area (S1) and contralateral dentate nucleus were identified. Additionally, volume of tissue activation (VTA) corresponding to active DBS contacts were modelled. Response to treatment was defined as 40% reduction in the total Fahn-Tolosa-Martin Tremor Rating Score (FTMTRS) with DBS-ON, one year from surgery. Three out of nine patients had a suboptimal, long-term response to treatment. The segmented thalamic areas corresponded well to anatomically known counterparts in the ventrolateral (VL) and ventroposterior (VP) thalamus. The dentate-thalamic area, lay within the M1-thalamic area in a ventral and lateral location. Streamlines corresponding to the DRT connected M1 to the contralateral dentate nucleus via the dentate-thalamic area, clearly crossing the midline in the mesencephalon. Good response was seen when the active contact VTA was in the thalamic area with highest connectivity to the contralateral dentate nucleus. Non-responders had active contact VTAs outside the dentate-thalamic area. We conclude that probabilistic tractography techniques can be used to segment the VL and VP thalamus based on cortical and cerebellar connectivity. The thalamic area, best representing the VIM, is connected to the contralateral dentate cerebellar nucleus. Connectivity based segmentation of the VIM can be achieved in individual patients in a clinically feasible timescale, using HARDI and high performance computing with parallel GPU processing. This same technique can map out the DRT tract with clear mesencephalic crossing.

## Highlights

• The thalamic target for surgery for tremor is not readily visible on conventional MRI.
• Probabilistic tractography is used to segment the thalamus based on connectivity.
• The best target area is connected to the contralateral dentate cerebellar nucleus.
• GPU processing is used to segment the thalamus in a clinically feasible timescale.
• This technique can map out the DRT tract with clear mesencephalic crossing.

## 1 Introduction

The ventral intermediate nucleus (VIM) of the thalamus is an established surgical target, for stereotactic ablation and deep brain stimulation (DBS) in the treatment of tremor in Parkinson's disease (PD), essential tremor (ET) and multiple sclerosis (Benabid et al., 1989, Benabid et al., 1991, Benabid et al., 1993; Berk et al., 2004; Hariz et al., 2007; Pahwa et al., 2001; Pollak et al., 1993; Schuurman et al., 2008). A subjacent area, the caudal zona incerta (cZI), is another effective DBS target for the treatment of tremor (Blomstedt et al., 2007, Blomstedt et al., 2009, Blomstedt et al., 2010; Murata et al., 2003; Plaha et al., 2008).

The VIM is centrally placed on a cerebello-thalamo-cortical network in which pathological oscillations, possibly triggered by pallidal dysfunction in the case of PD, is thought to be culpable for tremor (Helmich et al., 2011). The cortical focus in this tremor network is in the primary motor cortex, connected to the dentate nucleus of the contralateral cerebellum through the dentato-rubro-thalamic tract (DRT) via the VIM (Baker et al., 2010; Dum and Strick, 2003; Gallay et al., 2008; Helmich et al., 2012; Jörntell and Ekerot, 1999; McIntyre and Hahn, 2010).

The VIM is not readily visible on conventional, stereotactic MR imaging sequences used in image guided and image verified surgery (Deistung et al., 2013; Lemaire et al., 2010; Traynor et al., 2011; Vassal et al., 2012). Identifying the nucleus traditionally involves indirect targeting relying on atlas-defined coordinates in relation to the anterior commissure (AC) – posterior commissure (PC) points as landmarks, along with other identifiable structures such as the lateral thalamic/internal capsule border (Schaltenbrand et al., 1977). Needless to say, this approach does not fully account for individual variability. Furthermore, surgery often needs to be performed with the patient awake to allow for intraoperative confirmation of targeting, thus increasing patient discomfort (Gross et al., 2006). Moreover, intraoperative confirmation is not always readily feasible e.g. when performing a thalamotomy using Gamma Knife (Witjas et al., 2015) or focused ultrasound (Elias et al., 2016).

To overcome this, various imaging techniques have been proposed to identify the VIM. Ultra-high field MRI provides high contrast-to-noise ratio in-between thalamic nuclei, better segmenting the nucleus, however, this modality is not readily available in a clinical setting (Spiegelmann et al., 2006). Another technique relies on contrast in coloured fractional anisotropy (FA) maps, a product of diffusion tensor imaging (DTI) (Lefranc et al., 2015; Sedrak et al., 2011). Simple visualisation of the first order tensor fields in DTI has also been used to generate deterministic tractography models of the DRT, which is then targeted by DBS (Coenen et al., 2011, Coenen et al., 2014, Coenen et al., 2016; Sammartino et al., 2016). This modality is commonly accessible in clinical settings and imaging is relatively swift to acquire and process; however, it carries limitations related to disentangling crossing fibres, tracking in areas of low anisotropy (e.g. the thalamus) (Ramnani et al., 2004) and overall accuracy (Petersen et al., 2016).

An emerging modality utilises high angular resolution diffusion imaging (HARDI) and probabilistic connectivity based segmentation of the thalamus (Behrens et al., 2003a; Calabrese et al., 2015; Lambert et al., 2016; Miller et al., 2011; Ramnani et al., 2004). This technique successfully models crossing fibres and grey matter (low anisotropy) connectivity and achieves high signal-to-noise ratio, but requires prolonged image acquisition and large computational resources which are impractical in clinical practice. Novel MRI acquisition techniques, such as Simultaneous Multi-Slice Imaging and Multi-Band Imaging (Feinberg and Setsompop, 2013) have reduced scanning time. Furthermore, advances in computer processing techniques and relying on graphical processing units to carry out diffusion analysis have facilitated the use of this modality in clinical practice (Hernandez et al., 2013; Hernandez-Fernandez et al., 2016).

The objectives of this study were to examine the feasibility of using probabilistic, connectivity based segmentation techniques to segment the thalamus in a group of PD and ET patients one year from VIM DBS; to generate probabilistic tractography models of the DRT tracts and to carry out a post-hoc analysis of the relation of the segmented VIM and DRT with volume of tissue activation (VTA) models around active contacts of the DBS lead. We show that the VIM is best segmented based on connectivity to the contralateral dentate nucleus and that patients with good response to treatment had active contact VTAs within the segmented VIM.

## 2 Materials and methods

This study received ethical approval by West London NHS Research Ethics Committee (10/H0706/68). All participants provided written informed consent.

#### 2.1.1 Patients

Four male patients with tremor dominant PD who met UK brain bank criteria (Hughes et al., 1992) and five patients (3 female) with ET were recruited, following selection for VIM-DBS, by a multidisciplinary team of specialised movement disorders neurologists and functional neurosurgeons (Table 1). Formal neuropsychological assessment and structural brain MRI were performed to rule out dementia and significant brain atrophy, respectively. PD patients underwent the l-DOPA challenge test during the routine selection process. The motor subsection of the unified Parkinson's disease rating scale (UPDRS-III) was assessed in the OFF state at least 12 h after omitting PD medications. The assessment was then repeated 30 min (or when clinically ON) after administration of the patient's regular medications topped-up with an additional dose of 50 mg/12.5 mg dispersible Madopar/Benserazide. Patients with ET underwent assessment with the Fahn-Tolosa-Marin Tremor Rating Scale (FTMTRS) (Jankovic and Tolosa, 2007). The scale consists of three sections rating severity of tremor from 0 (none) to 4 (severe). The first section assesses severity and location of tremor, the second section assesses ability to perform specific motor tasks, such as writing, drawing and pouring, and the third section assesses patient-reported functional disability resulting from the tremor (speaking, eating, drinking, hygiene, dressing, writing, working and social activities) (Hess and Pullman, 2012). Inclusion in the present study was limited to patients who could tolerate lying flat for the duration of the preoperative scan and who have no contraindications to 3T MRI.

**Table 1:** Demographics, preoperative UPDRS-III (PD patients), FTMTRS (ET patients), postoperative FTMTRS ON/OFF DBS and stimulation parameters.

Patient		PD1	PD2	PD3	PD4	Mean	ET1	ET2	ET3	ET4	ET5	Mean
Age (yr.)*		67	63	64	67	65.3	56	49	66	78	70	63.8
Surgery		Left	Left	Bilat.	Left		Left	Left	Left	Left	Left	
Disease duration (yr.)*		5	6	10	10	7.8	10	10	6	12	11	9.8
Follow-up (month)		36	23	19	15	23.3	35	31	27	13	12	23.6
Preop. UPDRS-III tremor subsection (PD patients)	OFF MED.	12	8	17	13	12.5	–	–	–	–	–	
	ON MED.	12	8	11	8	9.8	–	–	–	–	–	
	IMP (%)	0 (0%)	0 (0%)	6 (35%)	5 (38.4%)	2.8 (18.4%)	–	–	–	–	–	
Preop. FTMTRS (ET patients)		–	–	–	–		55	66	93	97	97	81.6
Postop. FTMTRS	OFF DBS	32	33	129	55	62.3	44	71	93	89	63	72
	ON DBS	14	15	44	24	24.3	29	47	81	47	36	48
	IMP (%)	18 (56%)	18 (55%)	85 (66%)	31 (56%)	38 (58%)	15 (34%)	24 (34%)	24 (13%)	24 (47%)	24 (43%)	24 (34%)
ACTIVE CONTACTS	Left	1	2	2	0		1	0 plus 1	1	0 plus 3	3	
	Right	–	–	10	–		–	–	–	–	–	
	AMP (Volt)	2	2	2.6	1.8	2.1	2	2	2	2.5	2.5	2.2
	PW (μS)	60	60	60	60	60	60	60	60	60	60	60
	FREQ (HZ)	130	150	130	130	135	130	180	130	150	180	154

Yr.: year; IMP: improvement; AMP: amplitude; PW: pulse width; FREQ: frequency.

#### 2.1.2 Preoperative diffusion weighted MRI acquisition

Imaging was performed on a 3T Siemens Magnetom Trio TIM Syngo MR B17 using a 32 channel receive head coil. Concerted efforts to reduce head tremor were made by optimising drug therapy and using padding inside the head coil to reduce discomfort and head motion.

Siemens' 511E-Advanced Echo Planar Imaging Diffusion WIP was used. In-plane acceleration was used (GRAPPA factor of 2) with partial Fourier 6/8. In plane resolution was 1.5 × 1.5 mm^2^ (Field of view 219 × 219 mm^2^, TR = 12,200 ms, TE = 99.6 ms) and 85 slices were acquired with a 1.5 mm thickness. Diffusion-weighting, with b = 1500 s/mm^2^, was applied along 128-directions uniformly distributed on the sphere and seven b = 0 s volumes were also acquired. To correct for distortions, all acquisitions were repeated with a reversed phase encoding direction (left to right and right to left phase encode) giving a total of 270 volumes acquired ([128 + 7] × 2). Total acquisition time was 62 min.

#### 2.1.3 Surgical procedure and intraoperative MRI acquisition

DBS leads (3389 Medtronic) were implanted under local anaesthesia using a stereotactic MRI-guided and MRI-verified approach without microelectrode recording (using a Leksell frame model G, Elekta Instrument AB, Stockholm, Sweden), as detailed in previous publications on subthalamic nucleus DBS for PD (Foltynie et al., 2011; Holl et al., 2010). All patients had unilateral surgery except for one patient with PD tremor who underwent bilateral surgery.

Three stereotactic, pre-implantation scans were acquired, as part of the surgical procedure, to guide lead implantation; a proton-density and a T2 weighted axial scan (partial brain coverage around the thalamus and cZI) with voxel size of 1.0 × 1.0 mm^2^ and slice thickness of 2 mm (Hariz et al., 2003; Hirabayashi et al., 2002); and a T1 weighted 3D-MPRAGE scan with a 1.5 mm^3^ voxel size on a 1.5T Siemens Avanto interventional MRI scanner. Three-dimensional distortion correction was carried out using the scanner's built-in module. Once scans were reoriented to have slices parallel with the anterior commissure (AC) – posterior commissure (PC) line, the trajectory was planned such that the deepest contact targeted the cZI and the proximal contacts targeted the VIM at the level of the AC-PC. The thalamo-capsular border, visualised on the proton-density scan, was used to aid the identification of the laterality of the VIM on imaging, which was then indirectly targeted using atlas coordinates in relation to the mid-commissural point - [X = 12–14 mm, Y = (AC-PC length/3) − 2 mm anterior to PC, Z = 0]. The cZI was identified on the axial T2-weighted scan medial to the postero-medial border of the STN. The MPRAGE scan was used to plan the lead's entry point over the coronal suture ±1 cm anteroposteriorly, with the lead trajectory avoiding the ventricles and sulci. A 1.5 mm thick radiofrequency probe (RF) was inserted first into the deepest target (cZI), using impedance recording. The last 6 mm of the trajectory were traversed using 2 mm steps whilst simultaneously assessing the implantation effect on tremor in the outstretched contralateral upper limb. The RF lead was then replaced with the DBS lead, temporarily fixed in situ. Fibrin sealant (Tisseel, Baxter, USA) was used in the burr hole to prevent CSF leak and pneumocephalus (Petersen et al., 2010). An external stimulator was then used to deliver monopolar stimulation to each contact using increasing amplitudes to assess efficacy and side-effect profile. Transient tingling in the palm upon stimulation was considered a sign of good placement. Patients were stressed, using verbal recollection and arithmetic tasks, to elicit the tremor. Thresholds for capsular effects and dysesthesia were also assessed. In the case of poor response or unacceptable side-effects, the lead was removed and the process repeated following appropriate targeting adjustments. Imaging was repeated immediately following lead implantation to confirm lead placement. The specific absorption rate (SAR) was kept <0.4 W/kg by reducing the number of acquired T2 slices covering the distal leads to 12–14. The leads were then connected to an implantable pulse generator (IPG) (Activa SC or PC, Medtronic, Minneapolis, Minn., USA) implanted in the infra-clavicular region on the same day or within a week.

#### 2.1.4 Outcome measures

##### 2.1.4.1 Effective stimulation parameters

All DBS contacts were screened by a movement disorders neurologist once implantation effects had worn off (2–14 days). Patients were then regularly followed-up in clinic to adjust and fine tune stimulation in the first 12 months after surgery.

##### 2.1.4.2 Fahn-Tolosa-Marin tremor rating scale

All patients underwent assessment both in the OFF and ON DBS states 12–24 months from surgery. This was carried out by an experienced movement disorders neurologist. The assessment was carried out with DBS ON first and then 10 min after switching stimulation off. Good response to DBS was defined as an improvement ≥40% in total FTMTRS with ON stimulation.

##### 2.1.4.3 DBS contacts volume of tissue activated (VTA) modelling

SureTune® (Medtronic Inc. Minnesota), a DBS therapy planning platform was used to model VTAs around individual contacts. The platform applies neuron models coupled to finite element simulations as described by Åström and colleagues in order to generate DBS therapy VTA (Åström et al., 2015). Intraoperative MRI scans were uploaded and a two-step linear registration was used to co-register the pre-implantation and post-implantation stereotactic MPRAGE scans. The first step involved manually aligning the volumes with the pre-implantation MPRAGE. The second step employed automated co-registration with a restricted volume of fusion centred around the diencephalon/mesencephalon. This was carried out to minimise registration error resulting from eventual brain shift incurred during surgery, despite minimal brain shift with our surgical technique (Petersen et al., 2010). Registration accuracy was carefully inspected and the process iterated if necessary. All volumes were realigned with a plane parallel to the AC-PC line.

Post-implantation MPRAGE scans were used to fit the DBS lead model within the MRI artefact produced by the leads. Individual VTAs were then generated around active DBS contacts with corresponding stimulation amplitudes. Binary image files of VTAs with corresponding transformation matrices were exported and processed in Matlab (Mathworks Inc.) using an in-house software to generate Neuroimaging Informatics Technology Initiative (NIfTI) volumes for further analysis. Right sided individual contact VTAs were lateralised to the left by swapping the x axis (x, y, z > −x, y, z) using *Fslswapdim* (FSL v5.0).

#### 2.1.5 Image preprocessing

Pre-implantation MPRAGE scans were brain extracted using *BET* (Brain Extraction Tool, FSL v5.0) (Smith, 2002). Two-step transformation was used to register native scans to the MNI ICBM 152 non-linear (6th Generation) symmetric standard-space T1-weighted average structural template image (1 mm resolution) (Grabner et al., 2006). The first step employed linear (affine) transformation using *FLIRT* (FMRIB's Linear Image Registration Tool) using 12 degrees of freedom, correlation ratio cost function and normal search (Jenkinson et al., 2002; Jenkinson and Smith, 2001). The output from this step was used to execute non-linear registration (second step) using *FNIRT* (FMRIB's Non-Linear Image Registration Tool) (Andersson et al., 2007). This process produced individual native to standard (MNI space) non-linear warp fields, which were then applied to VTAs acquired from SureTune in order to transform all volumes to standard space.

#### 2.1.6 Diffusion pre-processing

Diffusion weighted imaging (DWI) scans (with accompanying b = 0 scans) were imported from DICOM (Digital Imaging and Communications in Medicine) files to NifTI volumes and the diffusion gradient direction values and vectors were extracted using *Volconv* (MJ White, NHNN Neuroradiology Department, London UK).

The diffusion data were acquired with reversed phase-encode blips (left-to-right and right-to-left), resulting in pairs of images with distortion going in opposite directions. From these pairs, the susceptibility-induced off-resonance field was estimated using a method described by (Andersson et al., 2003), as implemented in FSL (Smith et al., 2004) and the two images were combined into a single corrected one using *Topup* (FSL v5.0), a tool for estimating and correcting susceptibility induced distortions prevalent in SSEPI DWI. The output from *Topup* was then fed into Eddy (FSL v5.0) for correction of eddy current distortions and subject movement (Andersson and Sotiropoulos, 2016).

Patient averaged distortion corrected b = 0 volumes were registered to brain extracted structural images in native patient space (pre-implantation MPRAGE) with *Flirt* (FSL v5.0) using linear registration with six degrees of freedom, normal search and correlation ratio cost function. The resultant transformation matrices were then combined with the transformations previously generated using non-linear registration between the structural in native patient space and the standard MNI152-1mm symmetric space, producing diffusion-to-standard space transformations and their corresponding inverses.

*BedpostX* (FSL v5.0) was used to estimate fibre orientations. Up to three crossing fibres were estimated in each brain voxel using model 2 and graphics processing unit (GPU) parallelization (Hernandez et al., 2013; Jbabdi et al., 2012). Using the obtained transformations to and from standard space, tractography protocols and masks were defined in MNI space.

#### 2.1.7 Analysis

##### 2.1.7.1 Regions of interest (ROI) definition

**Fig. 1:**
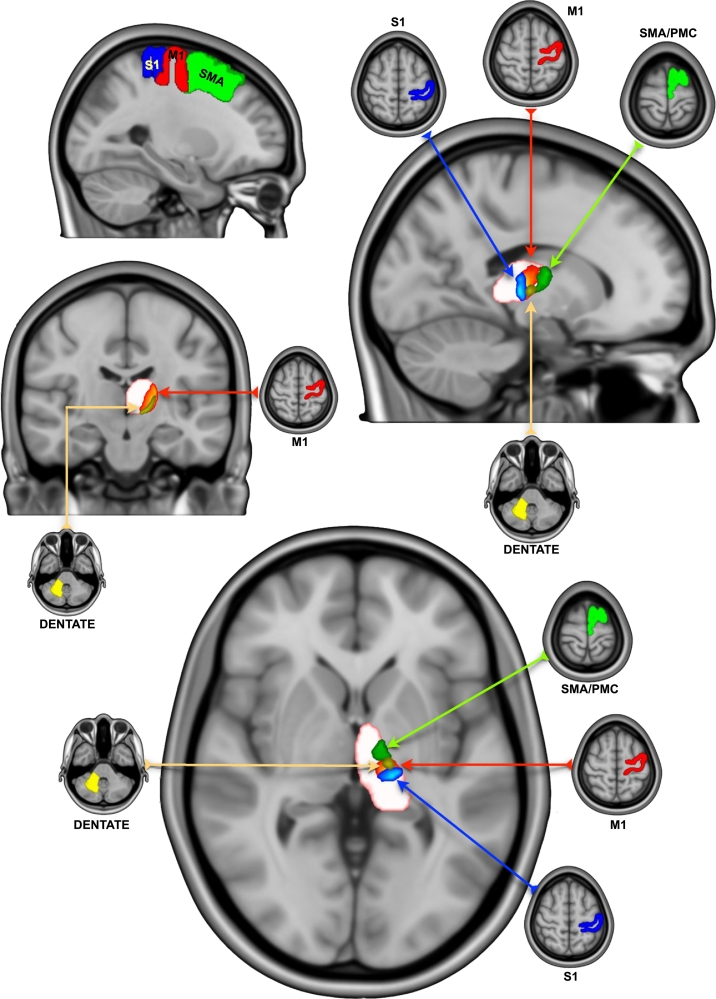
Thalamic clusters with corresponding cortical and cerebellar ROI masks (S1: blue - M1: red - SMA/PMC: green - dentate: yellow). S1: primary sensory area; M1: primary motor area; SMA: supplementary motor area; PMC: premotor cortex. (For interpretation of the references to color in this figure legend, the reader is referred to the web version of this article.)

Cortical reconstruction and volumetric segmentation of the MNI-152, symmetric T1 weighted (1 mm) volume was performed with the Freesurfer image analysis suite. Resulting ROIs were used for connectivity based thalamic segmentation and tractography of the dentato-rubro-thalamo-cortical tract (DRTC). The technical details of these procedures are described in prior publications (Fischl et al., 2002, Fischl et al., 2004). Cortical volumetric masks of the primary motor cortex (M1 [Brodmann's area 4]), primary sensory cortex (S1 [Brodmann's areas 3,1,2]), supplementary motor area (SMA), premotor cortex (PMC) (both constituting Brodmann's area 6) and subcortical thalamic volumetric masks were generated. Cerebellar masks of the superior cerebellar peduncle and the cerebellar white matter (containing the dentate nucleus) were manually segmented using ITK-SNAP (Yushkevich et al., 2006) (Fig. 1).

##### 2.1.7.2 Tractography

Probabilistic tractography was generated in ProbtrackX2 GPU version (Behrens et al., 2007) (Hernandez-Fernandez et al., 2016) (FSL v5.0) (number of samples = 5000, curvature-threshold = 0.2, step length = 0.5 mm subsidiary fibre volume fraction threshold = 0.01). The process repetitively samples from the distributions of voxel-wise principal diffusion directions generated in *BedpostX*, each time computing a streamline through these local samples to generate a ‘probabilistic streamline’ or a ‘sample’ from the distribution on the location of the true streamline, building up a spatial ‘connectivity distribution’ or global connectivity (i.e. the probability of the existence of a path through the diffusion field between any two distant points, a surrogate measure of anatomical connectivity) (Behrens et al., 2007). Streamlines truly represent paths of minimal hindrance to diffusion of water in the brain, but they are reasonable indirect estimates of long-range white matter connections (Jbabdi and Johansen-Berg, 2011).

##### 2.1.7.3 Connectivity based thalamic segmentation

Thalamic segmentation was carried out for all patients using probabilistic tractography. The resulting volumes were used to create group averages. Seed voxels in the thalamus were classified according to the probability of connection to the defined cortical and cerebellar target masks (ipsilateral S1, M1, SMA/PMC and contralateral cerebellar masks). This process has been previously described by Behrens et al. (Behrens et al., 2003a). CSF termination and contralateral cerebrum/ipsilateral cerebellum exclusion masks were applied to exclude false positive streamlines and commissural tracts.

##### 2.1.7.4 Tractography of the dentato-rubro-thalamo-cortical pathway

Probabilistic tractography was generated, for each patient, from the cerebellar seed to the contralateral M1 target using the contralateral thalamic mask as waypoint and the ipsilateral cerebrum and contralateral cerebellum as exclusion masks. CSF termination masks were used to exclude false positive streamlines. The process was repeated using the M1 mask as seed and the cerebellar mask as target. The two resulting tracks were merged to create a single DRTC tract. All tracks were then used to create group averages.

## 3 Results

### 3.1 Patients

Preoperative scanning and surgery proceeded with no adverse events. The mean pre-operative UPDDRS-III tremor subsection score (highest possible score = 28) for the PD patients was 12.5 (8–17) points off medications and 9.8 (8–12) points on medications with a modest average improvement of 18%. Two out of the four patients with PD did not show improvement in tremor with levodopa administration.

The ET group had a preoperative FTMTRS score of 81.6 (55–97) points.

All patients were right hand dominant. There was no surgical morbidity or mortality. One patient with PD had bilateral surgery in one procedure. The remainder had left sided surgery making up a total of 10 implanted DBS leads (five in each group) (Table 1).

### 3.2 DBS profile

All patients improved with DBS albeit to varying degrees (Table 1). PD3 had a marked improvement in tremor following lead implantation (bilateral DBS). The tremor re-emerged a week later just before IPG insertion. Once DBS was switched on there was a significant improvement in tremor, however, 24 h later, the patient became agitated. This was felt to be largely due to sleep deprivation and resolved on resumption of normal sleep. ET1 had a significant improvement at 2 V, however, mild slurring and slowing of speech occurred at 2.5 V. ET2 had tingling and discomfort in the right side of the face, right arm and part of the right leg when stimulating the deepest contact (cZI) and facial pulling at 1.9 V when stimulating the second deepest contact. ET4 developed very mild balance deterioration and a feeling of exhaustion and ET5 developed mild and transient paraesthesia with stimulation.

### 3.3 Postoperative clinical outcomes

All PD patients experienced tremor rebound when DBS was switched off. The mean improvement in FTMTRS was 58% in the PD group and 34% in the ET group, comparing off to on stimulation. Three out of five patients in the ET group had a poor response to treatment (<40%) (Table 1).

### 3.4 Connectivity-based thalamic segmentation

Appropriate thresholds of (1000) and (100) samples per voxel were applied to cortical and cerebellar group average thalamic clusters respectively using *Fslmaths* (FSL v5.0). The clusters were in the ventrolateral thalamus with some overlap between SMA/PMC and M1 clusters; and between M1 and S1 clusters. The contralateral cerebellar (dentate) cluster lies completely within the inferior portion of the M1 cluster (Fig. 1). Cluster-based inference using *Cluster* (FSL v5.0) was carried out to extract the clusters and local maxima in outputs (Table 2). The SMA, M1, S1 and dentate thalamic clusters are available to download online.

**Table 2:** Connectivity-based thalamic clusters of cortical and cerebellar areas showing volumes and MNI (AC-PC) coordinates of maximum intensity and centre of gravity (Left hemisphere).

Thalamic cluster	VOL (mm^3^)	Maximum intensity coordinates MNI (AC-PC)			Centre of gravity coordinates MNI (AC-PC)		
		X	Y	Z	X	Y	Z
S1	704	−17 (−16.5)	−23 (−11)	4 (8)	−17 (−16.5)	−22 (−10)	4.8 (8.8)
SMA/PMC	743	−15 (−14.5)	−8 (4)	5 (9)	−13 (−12.5)	−10 (2)	5.6 (9.6)
M1	1021	−20 (−19.5)	−19 (−7)	8 (12)	−16 (−15.5)	−19 (−7)	6 (10)
Dentate	141	−10 (−9.5)	−18 (−6)	−3 (1)	−15 (−14.5)	−17 (−5)	1.5 (5.5)

MNI: Montreal Neurological Institute; AC-PC: anterior commissure – posterior commissure; VOL: volume; S1: primary sensory area; M1: primary motor area; SMA: supplementary motor area; PMC: premotor cortex.

### 3.5 Tractography of the DRTC

**Fig. 2:**
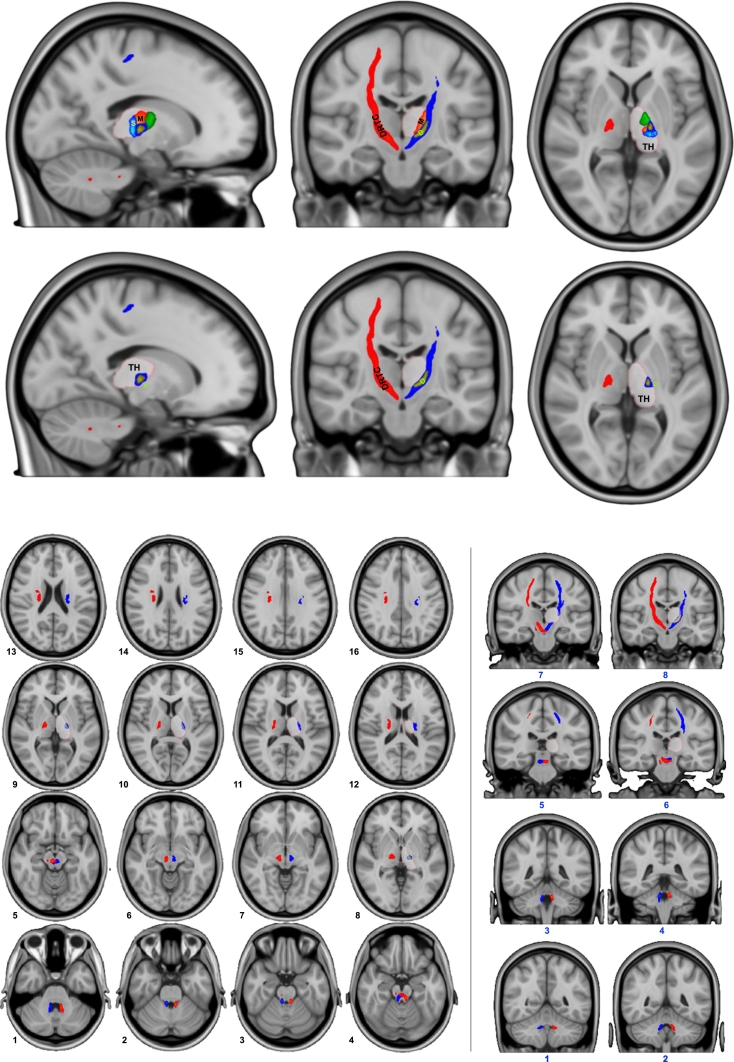
The left (blue) and right (red) dentato-rubro-thalamo-cortical tracts shown with decussation in the midbrain and path through the thalamic clusters. (For interpretation of the references to color in this figure legend, the reader is referred to the web version of this article.)

Left and right group average streamlines connect the dentate nucleus to the contralateral primary motor cortex, passing through the contralateral red nucleus and thalamus. The path through the thalamus clearly traverses the cerebellar cluster and overlapping portion of the M1 cluster (Fig. 2).

### 3.6 VTA modelling and relationship to thalamic clusters and DRTC

**Fig. 3:**
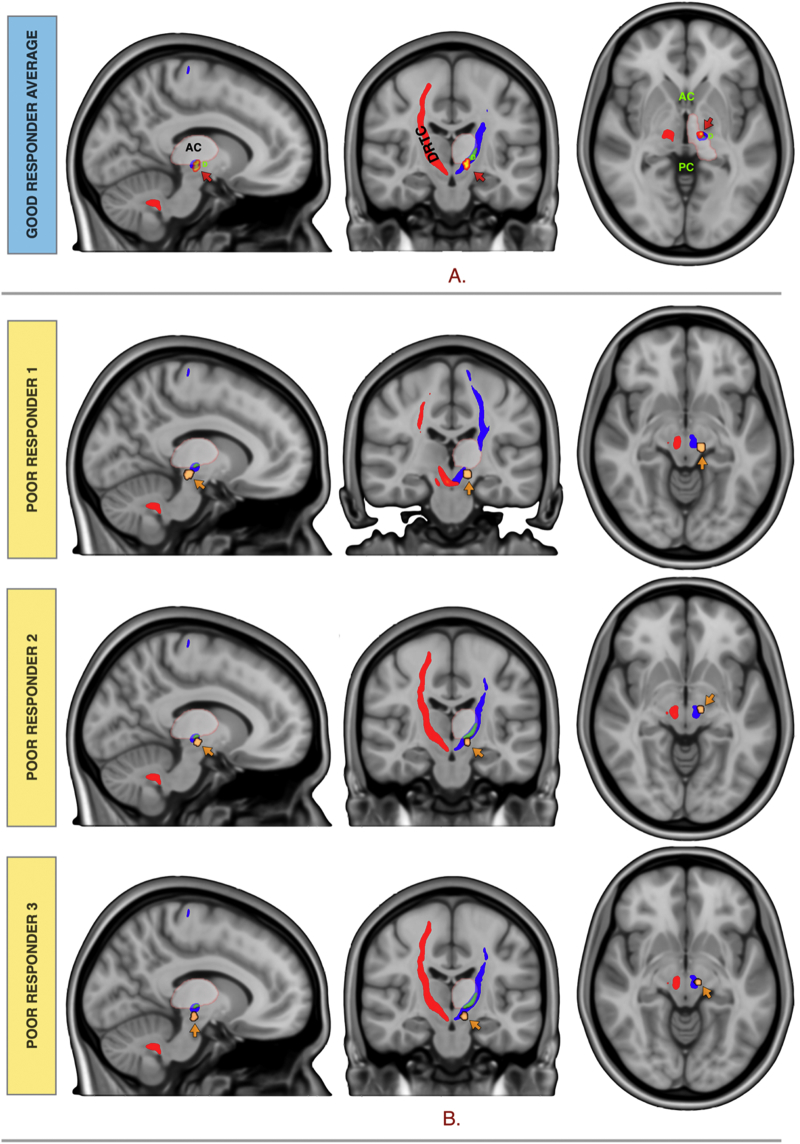
(A) Responders group average VTA (hot) and (B) non-responders VTAs (copper) in relation to the DRTC and the dentate-thalamic cluster.

VTA volumes corresponding to the active contacts stimulation for the seven patients with good response were averaged taking the median voxels. The good response group average fell on the dentate-thalamic cluster at the level of the AC-PC extending inferiorly into the cZI, on the DRTC. The three patients with poor response fell adjacent to, or on the DRTC but outside the dentate-thalamic cluster. See Fig. 3 for group average responders VTA and non-responders VTAs in relation to the DRTC and the dentate-thalamic cluster.

### 3.7 Group average dentate-thalamic cluster as an atlas template for surgical planning

Inverse, non-linear transformation warps were applied to the group average dentate-thalamic cluster using *ApplyWarp* (FSL v5.0). This reverse registration procedure was undertaken to compare dentate-thalamic clusters generated using patient-specific data to the cluster generated from the template. *Cluster* (FSL v5.0) toolbox was used to extract maximum intensity and centre of gravity voxel coordinates (in patient space) from patient-specific and template generated clusters. The average variance in the X,Y and Z coordinates between the two clusters was 1.5 mm (±1.2), 0.9 mm (±0.8) and 0.7 mm (±0.8) respectively for the maximum intensity and 0.6 mm (±0.6), 1.3 mm (±0.8) and 0.5 mm (±0.4) for the centre of gravity voxels. This meant that the average Euclidian distance between the two clusters was 2.2 mm (±1.2) for the maximum intensity and 1.6 mm (±0.9) for the centre of gravity voxels (Supplementary Table 1).

### 3.8 Feasibility of stereotactic DBS targeting of the dentate-thalamic cluster

**Fig. 4:**
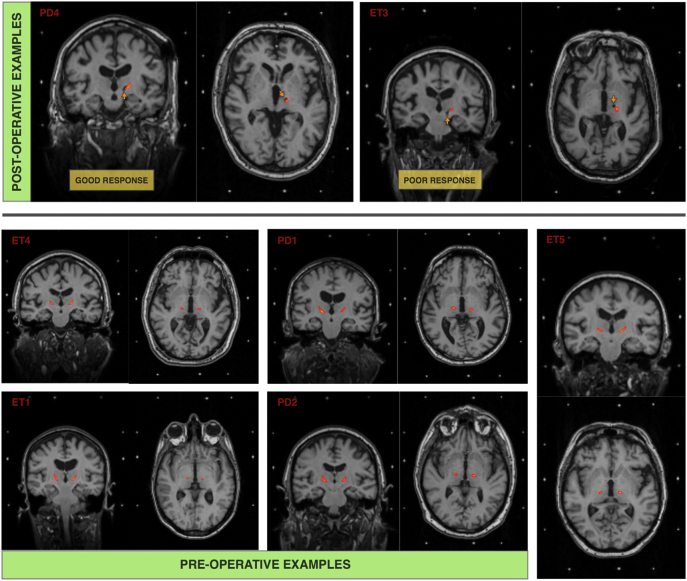
Individual dentate-thalamic clusters (red) registered to postoperative (top) and preoperative (bottom) stereotactic MPRAGE scans. (For interpretation of the references to color in this figure legend, the reader is referred to the web version of this article.)

Employing the methods described, segmentation and registration of the dentate-thalamic cluster was achieved in <10 min per subject using a local, purpose built GPU high performance computer with 10,752 Compute Unified Device Architecture (CUDA) cores. Diffusion preprocessing, using the same cluster, was achieved in under 45 min. See Fig. 4 for individual dentate-thalamic clusters registered to post- and preoperative stereotactic MPRAGE scans.

**Fig. 5:**
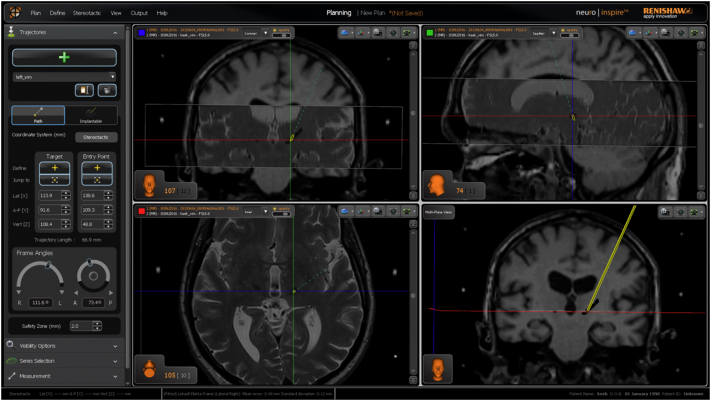
Left VIM DBS planning using Neuroinspire surgical planning software using preoperative stereotactic T2-weighted slab registered to MPRAGE T1 NIfTY volume with dentate-thalamic clusters punched-out

Neuroinspire™ surgical planning software (Renishaw PLC, United Kingdom) was used to carry out mock stereotactic targeting. The package has the capability of loading NIfTI volumes as well as DICOM image formats. The dentate-thalamic cluster voxels were subtracted (removed) from the stereotactic preoperative MPRAGE scan using *Fslmaths* (FSL V5.0). This process resulted in a new NIfTI volume with the clusters “punched out”. Planning was then carried out routinely with the added identification of the dentate-thalmic cluster, at the level of the AC-PC as demonstrated in Fig. 5.

## 4 Discussion

In this work, we segmented the VIM nucleus of the thalamus, using connectivity based probabilistic techniques, applied to individual HARDI datasets, in five patients with ET and four patients with PD tremor, one year from thalamic DBS. Furthermore, we generated probabilistic streamlines representing the DRTC tracts, clearly connecting the M1 area with the contralateral dentate nucleus of the cerebellum via the VIM showing clear crossing in the brainstem. Three out of nine patients had a suboptimal, long-term response to treatment as demonstrated on improvement on FTMTRS. Post-hoc analysis of active DBS-contacts VTA models, showed that a good response is seen when the VTA was in the segmented VIM.

Individualised, image guided and image verified targeting of the VIM has been a quest of many in the field of functional neurosurgery. Inter-individual variability in the VIM's location has been illustrated in several studies. This was clearly shown in a functional connectivity study that analysed resting state fMRI scans in 58 healthy subjects (Anderson et al., 2011). Considerable individual variability of atlas-based VIM targeting was again demonstrated in a study that examined the VIM's relation to surrounding major fibre tracts using deterministic tractography in 10 patients with thalamic DBS for ET (Anthofer et al., 2014). We have also demonstrated in this work that using a template-derived (group atlas) dentate-thalamic cluster registered to individual patients results in unacceptably large Euclidian error when compared to a patient-derived cluster. The average error was 2.2 mm and 1.6 mm for the maximum intensity and centre of gravity voxels respectively with an individual error of up to 4 mm in some cases. In our practice of deep brain stimulation surgery, we have a threshold of 1.5 mm targeting error beyond which we routinely relocate implanted leads intraoperatively.

In 2003, Behrens et al. published a report detailing the use of probabilistic tractography in delineating boundaries between different thalamic nuclei, based on connectivity patterns between the thalamus and various cortical areas (Behrens et al., 2003a). This was the first time probabilistic tractography was used to parcellate grey matter structures, obtaining the quality of results that traditional maximum-likelihood or streamline approaches have failed to produce (Jones et al., 1999). The resulting thalamic segmentation corresponded well with previous histological findings (Morel et al., 1997) and tracer studies in non-human primates (Jones, 2012; Jones and Powell, 1970; Jones et al., 1979; Markowitsch et al., 1987; Russchen et al., 1987; Tanaka, 1976; Tobias, 1975; Yarita et al., 1980). This technique was further validated in another study in 2004 (Johansen-Berg, 2004). Other grey matter structures have also been segmented with a similar approach (Chowdhury et al., 2013; Johansen-Berg et al., 2008; Lambert et al., 2011).

Several studies have since used probabilistic tractography to examine VIM connectivity to cortical and cerebellar areas (Groppa et al., 2014; Hyam et al., 2012; Klein et al., 2012), or to segment the VIM based on said connectivity (Pouratian et al., 2011). Others have used probabilistic tractography alongside multidimensional atlas data to improve thalamic target localization (Jakab et al., 2012). Interestingly, a post hoc analysis with connectivity based segmentation of six patients with bilateral VIM DBS showed the effective DBS contacts to be in the thalamic region with the highest probability of connection to the premotor and supplementary motor cortices (Pouratian et al., 2011). This goes against prior anatomical knowledge (Morel et al., 1997) and the consistent findings from other connectivity studies (Anderson et al., 2011; Groppa et al., 2014; Hyam et al., 2012; Klein et al., 2012) and transcranial magnetic stimulation (TMS) studies (Ni et al., 2010). It is likely that this inconsistency resulted from using diffusion MR acquisition parameters intended for conventional clinical applications, such as mapping major white matter tracts prior to surgical intervention with low angular resolution (number of diffusion directions = 20), low spatial resolution (isotropic voxel size = 2 mm) and low angular contrast (b-value = 1000 s/mm^2^) (Pouratian et al., 2011).

Choosing the appropriate diffusion imaging parameters is paramount to achieving accurate segmentation of grey matter structures such as the thalamus (Calabrese et al., 2015; Lambert et al., 2016; Miller et al., 2011). In vivo probabilistic tractography studies in the cerebellum, brainstem and diencephalon carry significant challenges. Motion artefacts, caused by the highly pulsatile nature of the region, can degrade the MRI signal during diffusion image acquisition, reducing the signal-to-noise ratio (SNR). This is complicated by the presence of myriad criss-crossing axons and reticular brain regions (Lambert et al., 2013a, Lambert et al., 2013b). One way of dealing with this is by using pulse-gating and respiratory rate monitoring during diffusion imaging. Likewise, by acquiring multiple diffusion scans, at a high angular resolution (increasing acquisition time), SNR is improved (Behrens et al., 2003b, Behrens et al., 2007).

We acquired 270 diffusion scans per patient (in 2 × 128 directions sets) over 62 min. We meticulously and systematically corrected artefacts and examined the processed imaging data for quality control. We modelled three crossing fibres per voxel and used probabilistic tractography to ameliorate difficulties posed by crossing or kissing fibres and tunnelling effect (Behrens et al., 2007; Dyrby et al., 2007). To keep the analysis focused, a set of tractography rules based on knowledge from anatomical studies was used, without being too restrictive.

Our analysis shows that the thalamic area, with highest connectivity to the contralateral dentate nucleus lies within the much larger area with highest connectivity to M1 in a ventro-lateral position. The area with highest connectivity to the SMA and PMC was anterior to the M1 area. The area with highest connectivity to S1 was posterior to the M1 area. This is in keeping with known anatomical information (Nieuwenhuys et al., 2013). The ventral posterior (VP) thalamic nuclear complex relays impulses of sensory systems to S1, whilst ventral lateral (VL) nuclear complex relays information from the cerebellum, basal ganglia and substantia nigra (SN) (Nieuwenhuys et al., 2013). The VL complex is generally subdivided into the pars anterior (VLa), pars posterior (VLp) and pars medialis (VLm). The VLa relays afferents from the globus pallidus interna (Gpi) to the PMC and SMA (DeVito and Anderson, 1982; Kuo and Carpenter, 1973; Nauta, 1979; Parent and De Bellefeuille, 1982; Sakai et al., 1999, Sakai et al., 2000; Schaltenbrand et al., 1977); whilst the VLm relays input from the SN to the PMC and prefrontal cortex (Jones, 2012; Schell and Strick, 1984; Strick, 1973). The VLp, receives a large, topographically organised input from the cerebellar nuclei, projecting principally to M1 (Asanuma et al., 1983; Nieuwenhuys et al., 2013; Percheron et al., 1993; Sakai et al., 1999, Sakai et al., 2000). The VIM corresponds to the inferior part of the VLp (Jones, 2012).

It is important to bear in mind that the subdivisions of the thalamus by (Hassler, 1950) or (Hirai and Jones, 1989) are primarily based on histochemical staining of serial sections of human thalami, rather than anatomical connectivity. It is entirely possible that the optimal “functional” target straddles these subdivisions. Moreover, it is mechanistically likely that network connectivity of the target area will be a better predictor of efficacy than its histochemical properties.

Previous work focused on relation of DBS contacts to areas with cortical connectivity rather than cerebellar connectivity (Pouratian et al., 2011). We have shown these areas to be non-specific and with varying degrees of overlap. Whilst the dentate-thalamic area is more representative of the actual VIM, it is harder to segment due to inherent difficulties in diffusion connectivity techniques highlighted above. This is, to our knowledge, the first time such parcellation has been made possible, on the individual level, using in vivo 3T MRI.

Deterministic approaches have so far failed to produce anatomically accurate representations of the DRTC, generally showing the tract to arise from the ipsilateral, not the contralateral dentate nucleus (Coenen et al., 2011, Coenen et al., 2014, Coenen et al., 2016), or stopping at the upper brainstem decussation level (Sammartino et al., 2016). This may not be problematic when the DRTC itself is being targeted, as it is the case in these reports; however, to accurately segment the VIM based on cerebellar connectivity, the crossing cerebellar streamlines must be mapped. We show clear crossing of the DRTC from the contralateral dentate nucleus, which passes through the segmented dentate area in the thalamus all the way to M1. The average VTA of the responders group lies in the inferior dentate thalamic area and on the DRTC in the CZi, possibly capturing the DRTC fibres as they enter the VIM.

### 4.1 Using the FTMTRS as an outcome measure

Despite the prevalence of tremor amongst movement disorders, there is no universally accepted method of quantifying and rating its severity (Deuschl and Elble, 2000; Deuschl et al., 2000, Deuschl et al., 2001; Hess and Pullman, 2012; Jankovic and Tolosa, 2007). Several tremor scales exist but they are often disease specific (Elble et al., 2012). In 2013, a task force established by the Movement Disorders Society reviewed several rating scales for the assessment of tremor and recommended the use of five severity scales, one of which was the FTMTRS. The scale was assessed for reliability, validity and sensitivity to change (Elble et al., 2013). Moreover, in view of the mixed patient group in this study, the FTMTRS has the advantage of being non-disease specific (Stacy et al., 2007).

In this study, we examined the change in FTMTRS with DBS-OFF and -ON, 12–24 months from surgery. We did not calculate the improvement in FTMTRS in relation to preoperative baseline scores. This was since FTMTRS scores were not part of the routine preoperative assessment for PD patients which is a limitation of this study. It is interesting to notice the apparent ‘lesion effect’ in three out of five ET patients (two responders and one non-responder) illustrated by reduction in FTMTRS in the postoperative DBS-OFF measurements when compared to baseline. Indeed, the overall percentage of improvement with DBS-ON would have been higher had the preoperative FTMTRS been used as a denominator in the ET group.

Our results show that the patients in the PD group had a greater reduction in the average FTMTRS score with DBS. This can be attributed to the differences in the underlying pathology in ET and PD tremor (Deuschl et al., 2000; Deuschl and Elble, 2000) leading to a different response to DBS (Cury et al., 2017).

## 5 Limitations

In this study, a patient specific, finite element model was used to create DBS volumes of tissue activated (Åström et al., 2008). This is a simplified linear model that does not account for local impedance inhomogeneity. While we think it is important that efforts are put into improving models of DBS to resemble reality as much as possible, it may not help to add details to a rough model when the basic knowledge of the DBS mechanisms of actions are still debated. Indeed, various models over- or under-estimate the VTA (Maks et al., 2009). The presence of axons of different diameters and cell bodies, with variable action-potential thresholds, in the DBS region, complicates matters further.

By employing multiple registration steps, we have introduced error to the system. Nonetheless; we meticulously confirmed registration accuracy at each step to alleviate the impact of this issue.

Lastly, the relatively long scan duration is a drawback. This was accepted to achieve the required SNR and resolution. However, since this study was conducted, novel MRI acquisition techniques, such as Simultaneous Multi-Slice Imaging and Multi-Band Imaging (Feinberg and Setsompop, 2013) have been developed that will allow future studies to run similar protocols within half the time without compromising the SNR. Further improvements in diffusion imaging, with higher spatial and angular resolution, better MRI gradients and shorter acquisition times with emergence of multi-band acquisition will add to the value of this modality (Jbabdi and Johansen-Berg, 2011; Sotiropoulos et al., 2013). Lastly, the number of patients in this study is relatively small with mixed aetiologies. However, the data suggest that imaging can be used to optimise efficacy of tremor control. We intend to expand our experience with this technique in each pathology in the coming years.

## 6 Conclusion

Probabilistic tractography techniques can be used to segment the VL and VP thalamus based on cortical and cerebellar connectivity. The thalamic area, best representing the VIM, is connected to the contralateral dentate cerebellar nucleus. Patients with VTAs in this area attained good treatment response, whilst those with VTAs outside it did not. Connectivity based segmentation of the VIM can be achieved in individual patients in a clinically feasible timescale, using HARDI and high-performance computing with parallel GPU processing. This same technique can map out the DRTC with clear mesencephalic crossing. We advocate using patient-specific, connectivity-derived VIM in surgical targeting over non-specific, template-derived VIM due to unacceptable localisation error margin found in this study. Future studies may focus on improving data acquisition and processing time; and apply this technique prospectively in patients undergoing thalamic DBS or lesioning for tremor.

The following are the supplementary data related to this article.

Supplementary Table 1 Coordinates of template generated and patient specific dentate-thalamic clusters. Supplementary Table 1

Supplementary data NIfTI format thalamic segmentation imaging files of group average, connectivity derived S1, M1, SMA and contralateral cerebellar dentate regions Image 1

## References

[bb0005] Anderson J.S., Dhatt H.S., Ferguson M.A., Lopez-Larson M., Schrock L.E., House P.A., Yurgelun-Todd D. (2011). Functional connectivity targeting for deep brain stimulation in essential tremor. Am. J. Neuroradiol..

[bb0010] Andersson J.L.R., Sotiropoulos S.N. (2016). An integrated approach to correction for off-resonance effects and subject movement in diffusion MR imaging. NeuroImage.

[bb0015] Andersson J.L.R., Skare S., Ashburner J. (2003). How to correct susceptibility distortions in spin-echo echo-planar images: application to diffusion tensor imaging. NeuroImage.

[bb0020] Andersson J.L.R., Jenkinson M., Smith S. (2007). Non-linear Registration Aka Spatial Normalisation [WWW Document]. http://www.fmrib.ox.ac.uk/analysis/techrep/tr07ja2/tr07ja2.pdf.

[bb0025] Anthofer J., Steib K., Fellner C., Lange M., Brawanski A., Schlaier J. (2014). The variability of atlas-based targets in relation to surrounding major fibre tracts in thalamic deep brain stimulation. Acta Neurochir..

[bb0030] Asanuma C., Thach W.T., Jones E.G. (1983). Distribution of cerebellar terminations and their relation to other afferent terminations in the ventral lateral thalamic region of the monkey. Brain Res..

[bb0035] Åström M., Zrinzo L.U., Tisch S., Tripoliti E., Hariz M.I., Wårdell K. (2008). Method for patient-specific finite element modeling and simulation of deep brain stimulation. Med. Biol. Eng. Comput..

[bb0040] Åström M., Diczfalusy E., Martens H., Wårdell K. (2015). Relationship between neural activation and electric field distribution during deep brain stimulation. I.E.E.E. Trans. Biomed. Eng..

[bb0045] Baker K.B., Schuster D., Cooperrider J., Machado A.G. (2010). Deep brain stimulation of the lateral cerebellar nucleus produces frequency-specific alterations in motor evoked potentials in the rat in vivo. Exp. Neurol..

[bb0050] Behrens T.E.J., Johansen-Berg H., Woolrich M.W., Smith S.M., Wheeler-Kingshott C.A.M., Boulby P.A., Barker G.J., Sillery E.L., Sheehan K., Ciccarelli O., Thompson A.J., Brady J.M., Matthews P.M. (2003). Non-invasive mapping of connections between human thalamus and cortex using diffusion imaging. Nat. Neurosci..

[bb0055] Behrens T.E.J., Woolrich M.W., Jenkinson M., Johansen-Berg H., Nunes R.G., Clare S., Matthews P.M., Brady J.M., Smith S.M. (2003). Characterization and propagation of uncertainty in diffusion-weighted MR imaging. Magn. Reson. Med..

[bb0060] Behrens T.E.J., Berg H.J., Jbabdi S., Rushworth M.F.S., Woolrich M.W. (2007). Probabilistic diffusion tractography with multiple fibre orientations: what can we gain?. NeuroImage.

[bb0065] Benabid A.L., Pollak P., Hommel M., Gaio J.M., de Rougemont J., Perret J. (1989). Treatment of Parkinson tremor by chronic stimulation of the ventral intermediate nucleus of the thalamus. Rev. Neurol. (Paris).

[bb0070] Benabid A.L., Pollak P., Gervason C., Hoffmann D., Gao D.M., Hommel M., Perret J.E., de Rougemont J. (1991). Long-term suppression of tremor by chronic stimulation of the ventral intermediate thalamic nucleus. Lancet.

[bb0075] Benabid A.L., Pollak P., Seigneuret E., Hoffmann D., Gay E., Perret J. (1993). Chronic VIM thalamic stimulation in Parkinson's disease, essential tremor and extra-pyramidal dyskinesias. Acta Neurochir. Suppl. (Wien).

[bb0080] Berk C., Carr J., Sinden M., Martzke J., Honey C.R. (2004). Assessing tremor reduction and quality of life following thalamic deep brain stimulation for the treatment of tremor in multiple sclerosis. J. Neurol. Neurosurg. Psychiatry.

[bb0085] Blomstedt P., Hariz G.M., Hariz M.I., Koskinen L.O.D. (2007). Thalamic deep brain stimulation in the treatment of essential tremor: a long-term follow-up. Br. J. Neurosurg..

[bb0090] Blomstedt P., Sandvik U., Fytagoridis A., Tisch S. (2009). The posterior subthalamic area in the treatment of movement disorders: past, present, and future. Neurosurgery.

[bb0095] Blomstedt P., Sandvik U., Tisch S. (2010). Deep brain stimulation in the posterior subthalamic area in the treatment of essential tremor. Mov. Disord..

[bb0100] Calabrese E., Hickey P., Hulette C., Zhang J., Parente B., Lad S.P., Johnson G.A. (2015). Postmortem diffusion MRI of the human brainstem and thalamus for deep brain stimulator electrode localization. Hum. Brain Mapp..

[bb0105] Chowdhury R., Lambert C., Dolan R.J., Düzel E. (2013). Parcellation of the human substantia nigra based on anatomical connectivity to the striatum. NeuroImage.

[bb0110] Coenen V.A., Mädler B., Schiffbauer H., Urbach H., Allert N. (2011). Individual fiber anatomy of the subthalamic region revealed with diffusion tensor imaging: a concept to identify the deep brain stimulation target for tremor suppression. Neurosurgery.

[bb0115] Coenen V.A., Allert N., Paus S., Kronenburger M., Urbach H., Mädler B. (2014). Modulation of the cerebello-thalamo-cortical network in thalamic deep brain stimulation for tremor: a diffusion tensor imaging study. Neurosurgery.

[bb0120] Coenen V.A., Rijntjes M., Prokop T., Piroth T., Amtage F., Urbach H., Reinacher P.C. (2016). One-pass deep brain stimulation of dentato-rubro-thalamic tract and subthalamic nucleus for tremor-dominant or equivalent type Parkinson's disease. Acta Neurochir..

[bb0125] Cury R.G., Fraix V., Castrioto A., Pérez Fernández M.A., Krack P., Chabardes S., Seigneuret E., Alho E.J.L., Benabid A.L., Moro E. (2017). Thalamic deep brain stimulation for tremor in Parkinson disease, essential tremor, and dystonia. Neurology.

[bb0130] Deistung A., Schäfer A., Schweser F., Biedermann U., Turner R., Reichenbach J.R. (2013). Toward in vivo histology: a comparison of quantitative susceptibility mapping (QSM) with magnitude-, phase-, and R2⁎-imaging at ultra-high magnetic field strength. NeuroImage.

[bb0135] Deuschl G., Elble R.J. (2000). The pathophysiology of essential tremor. Neurology.

[bb0140] Deuschl G., Raethjen J., Baron R., Lindemann M., Wilms H., Krack P. (2000). The pathophysiology of parkinsonian tremor: a review. J. Neurol..

[bb0145] Deuschl G., Raethjen J., Lindemann M., Krack P. (2001). The pathophysiology of tremor. Muscle Nerve.

[bb0150] DeVito J.L., Anderson M.E. (1982). An autoradiographic study of efferent connections of the globus pallidus in Macaca mulatta. Exp. Brain Res..

[bb0155] Dum R.P., Strick P.L. (2003). An unfolded map of the cerebellar dentate nucleus and its projections to the cerebral cortex. J. Neurophysiol..

[bb0160] Dyrby T.B., Søgaard L.V., Parker G.J., Alexander D.C., Lind N.M., Baaré W.F.C., Hay-Schmidt A., Eriksen N., Pakkenberg B., Paulson O.B., Jelsing J. (2007). Validation of in vitro probabilistic tractography. NeuroImage.

[bb0165] Elble R., Comella C., Fahn S., Hallett M., Jankovic J., Juncos J.L., Lewitt P., Lyons K., Ondo W., Pahwa R., Sethi K., Stover N., Tarsy D., Testa C., Tintner R., Watts R., Zesiewicz T. (2012). Reliability of a new scale for essential tremor. Mov. Disord..

[bb0170] Elble R., Bain P., Forjaz M.J., Haubenberger D., Testa C., Goetz C.G., Leentjens A.F.G., Martínez-Martín P., Pavy-Le Traon A., Post B., Sampaio C., Stebbins G.T., Weintraub D., Schrag A. (2013). Task force report: scales for screening and evaluating tremor: critique and recommendations. Mov. Disord..

[bb0175] Elias W.J., Lipsman N., Ondo W.G., Ghanouni P., Kim Y.G., Lee W., Schwartz M., Hynynen K., Lozano A.M., Shah B.B., Huss D., Dallapiazza R.F., Gwinn R., Witt J., Ro S., Eisenberg H.M., Fishman P.S., Gandhi D., Halpern C.H., Chuang R., Butts Pauly K., Tierney T.S., Hayes M.T., Cosgrove G.R., Yamaguchi T., Abe K., Taira T., Chang J.W. (2016). A randomized trial of focused ultrasound thalamotomy for essential tremor. N. Engl. J. Med..

[bb0180] Feinberg D.A., Setsompop K. (2013). Ultra-fast MRI of the human brain with simultaneous multi-slice imaging. J. Magn. Reson..

[bb0185] Fischl B., Salat D.H., Busa E., Albert M., Dieterich M., Haselgrove C., van der Kouwe A., Killiany R., Kennedy D., Klaveness S., Montillo A., Makris N., Rosen B., Dale A.M. (2002). Whole brain segmentation: automated labeling of neuroanatomical structures in the human brain. Neuron.

[bb0190] Fischl B., van der Kouwe A., Destrieux C., Halgren E., Ségonne F., Salat D.H., Busa E., Seidman L.J., Goldstein J., Kennedy D., Caviness V., Makris N., Rosen B., Dale A.M. (2004). Automatically parcellating the human cerebral cortex. Cereb. Cortex.

[bb0195] Foltynie T., Zrinzo L., Martinez-Torres I., Tripoliti E., Petersen E., Holl E., Aviles-Olmos I., Jahanshahi M., Hariz M., Limousin P. (2011). MRI-guided STN DBS in Parkinson's disease without microelectrode recording: efficacy and safety. J. Neurol. Neurosurg. Psychiatry.

[bb0200] Gallay M.N., Jeanmonod D., Liu J., Morel A. (2008). Human pallidothalamic and cerebellothalamic tracts: anatomical basis for functional stereotactic neurosurgery. Brain Struct. Funct..

[bb0205] Grabner G., Janke A.L., Budge M.M., Smith D., Pruessner J., Collins D.L. (2006). Symmetric atlasing and model based segmentation: an application to the hippocampus in older adults. Med. Image Comput. Comput. Assist. Interv..

[bb0210] Groppa S., Herzog J., Falk D., Riedel C., Deuschl G., Volkmann J. (2014). Physiological and anatomical decomposition of subthalamic neurostimulation effects in essential tremor. Brain.

[bb0215] Gross R.E., Krack P., Rodriguez-Oroz M.C., Rezai A.R., Benabid A.L. (2006). Electrophysiological mapping for the implantation of deep brain stimulators for Parkinson's disease and tremor. Mov. Disord..

[bb0220] Hariz M.I., Krack P., Melvill R., Jorgensen J.V., Hamel W., Hirabayashi H., Lenders M., Wesslen N., Tengvar M., Yousry T.A. (2003). A quick and universal method for stereotactic visualization of the subthalamic nucleus before and after implantation of deep brain stimulation electrodes. Stereotact. Funct. Neurosurg..

[bb0225] Hariz M.I., Krack P., Alesch F., Augustinsson L.-E., Bosch A., Ekberg R., Johansson F., Johnels B., Meyerson B.A., Nguyen J.P., Pinter M., Pollak P., von Raison F., Rehncrona S., Speelman J.D., Sydow O., Benabid A.L. (2007). Multicentre European study of thalamic stimulation for parkinsonian tremor: a 6 year follow-up. J. Neurol. Neurosurg. Psychiatry.

[bb0230] Hassler R. (1950). Anatomy of the thalamus. Arch. Psychiatr. Nervenkr. Z Gesamte. Neurol. Psychiatr..

[bb0235] Helmich R.C., Janssen M.J.R., Oyen W.J.G., Bloem B.R., Toni I. (2011). Pallidal dysfunction drives a cerebellothalamic circuit into Parkinson tremor. Ann. Neurol..

[bb0240] Helmich R.C., Hallett M., Deuschl G., Toni I., Bloem B.R. (2012). Cerebral causes and consequences of parkinsonian resting tremor: a tale of two circuits?. Brain.

[bb0245] Hernandez M., Guerrero G.D., Cecilia J.M., García J.M., Inuggi A., Jbabdi S., Behrens T.E.J., Sotiropoulos S.N. (2013). Accelerating fibre orientation estimation from diffusion weighted magnetic resonance imaging using GPUs. PLoS One.

[bb0250] Hernandez-Fernandez M., Reguly I., Giles M., Jbabdi S., Smith S., Sotiropoulos S. (2016). A fast and flexible toolbox for tracking brain connections in diffusion MRI datasets using GPUs. Presented at the Organization for Human Brain Mapping (OHBM), Geneva, Switzerland.

[bb0255] Hess C.W., Pullman S.L. (2012). Tremor: clinical phenomenology and assessment techniques. Tremor Other Hyperkinet Mov (N Y).

[bb0260] Hirabayashi H., Tengvar M., Hariz M.I. (2002). Stereotactic imaging of the pallidal target. Mov. Disord..

[bb0265] Hirai T., Jones E.G. (1989). A new parcellation of the human thalamus on the basis of histochemical staining. Brain Res. Brain Res. Rev..

[bb0270] Holl E.M., Petersen E.A., Foltynie T., Martinez-Torres I., Limousin P., Hariz M.I., Zrinzo L. (2010). Improving targeting in image-guided frame-based deep brain stimulation. Neurosurgery.

[bb0275] Hughes A.J., Daniel S.E., Kilford L., Lees A.J. (1992). Accuracy of clinical diagnosis of idiopathic Parkinson's disease: a clinico-pathological study of 100 cases. J. Neurol. Neurosurg. Psychiatry.

[bb0280] Hyam J.A., Owen S.L.F., Kringelbach M.L., Jenkinson N., Stein J.F., Green A.L., Aziz T.Z. (2012). Contrasting connectivity of the ventralis intermedius and ventralis oralis posterior nuclei of the motor thalamus demonstrated by probabilistic tractography. Neurosurgery.

[bb0285] Jakab A., Blanc R., Berényi E.L., Székely G. (2012). Generation of individualized thalamus target maps by using statistical shape models and thalamocortical tractography. Am. J. Neuroradiol..

[bb0290] Jankovic J., Tolosa E. (2007). Parkinson's Disease and Movement Disorders.

[bb0295] Jbabdi S., Johansen-Berg H. (2011). Tractography: where do we go from here?. Brain Connect..

[bb0300] Jbabdi S., Sotiropoulos S.N., Savio A.M., Graña M., Behrens T.E.J. (2012). Model-based analysis of multishell diffusion MR data for tractography: how to get over fitting problems. Magn. Reson. Med..

[bb0305] Jenkinson M., Smith S. (2001). A global optimisation method for robust affine registration of brain images. Med. Image Anal..

[bb0310] Jenkinson M., Bannister P., Brady M., Smith S. (2002). Improved optimization for the robust and accurate linear registration and motion correction of brain images. NeuroImage.

[bb0315] Johansen-Berg H. (2004). Functional-anatomical validation and individual variation of diffusion tractography-based segmentation of the human thalamus. Cereb. Cortex.

[bb0320] Johansen-Berg H., Gutman D.A., Behrens T.E.J., Matthews P.M., Rushworth M.F.S., Katz E., Lozano A.M., Mayberg H.S. (2008). Anatomical connectivity of the subgenual cingulate region targeted with deep brain stimulation for treatment-resistant depression. Cereb. Cortex.

[bb0325] Jones E.G. (2012). The Thalamus.

[bb0330] Jones E.G., Powell T.P. (1970). Connexions of the somatic sensory cortex of the rhesus monkey. 3. Thalamic connexions. Brain.

[bb0335] Jones E.G., Wise S.P., Coulter J.D. (1979). Differential thalamic relationships of sensory-motor and parietal cortical fields in monkeys. J. Comp. Neurol..

[bb0340] Jones D.K., Simmons A., Williams S.C., Horsfield M.A. (1999). Non-invasive assessment of axonal fiber connectivity in the human brain via diffusion tensor MRI. Magn. Reson. Med..

[bb0345] Jörntell H., Ekerot C.F. (1999). Topographical organization of projections to cat motor cortex from nucleus interpositus anterior and forelimb skin. J. Physiol..

[bb0350] Klein J.C., Barbe M.T., Seifried C., Baudrexel S., Runge M., Maarouf M., Gasser T., Hattingen E., Liebig T., Deichmann R., Timmermann L., Weise L., Hilker R. (2012). The tremor network targeted by successful VIM deep brain stimulation in humans. Neurology.

[bb0355] Kuo J.S., Carpenter M.B. (1973). Organization of pallidothalamic projections in the rhesus monkey. J. Comp. Neurol..

[bb0360] Lambert C., Zrinzo L., Nagy Z., Lutti A., Hariz M., Foltynie T., Draganski B., Ashburner J., Frackowiak R. (2011). Confirmation of functional zones within the human subthalamic nucleus: patterns of connectivity and sub-parcellation using diffusion weighted imaging. NeuroImage.

[bb0365] Lambert C., Chowdhury R., Fitzgerald T.H.B., Fleming S.M., Lutti A., Hutton C., Draganski B., Frackowiak R., Ashburner J. (2013). Characterizing aging in the human brainstem using quantitative multimodal MRI analysis. Front. Hum. Neurosci..

[bb0370] Lambert C., Lutti A., Helms G., Frackowiak R., Ashburner J. (2013). Multiparametric brainstem segmentation using a modified multivariate mixture of Gaussians. Neuroimage Clin..

[bb0375] Lambert C., Simon H., Colman J., Barrick T.R. (2016). Defining thalamic nuclei and topographic connectivity gradients in vivo. NeuroImage.

[bb0380] Lefranc M., Carron R., Régis J. (2015). Prelemniscal radiations: a new reliable landmark of the thalamic nucleus ventralis intermedius location. Stereotact. Funct. Neurosurg..

[bb0385] Lemaire J.-J., Sakka L., Ouchchane L., Caire F., Gabrillargues J., Bonny J.-M. (2010). Anatomy of the human thalamus based on spontaneous contrast and microscopic voxels in high-field magnetic resonance imaging. Neurosurgery.

[bb0390] Maks C.B., Butson C.R., Walter B.L., Vitek J.L., McIntyre C.C. (2009). Deep brain stimulation activation volumes and their association with neurophysiological mapping and therapeutic outcomes. J. Neurol. Neurosurg. Psychiatry.

[bb0395] Markowitsch H.J., Irle E., Emmans D. (1987). Cortical and subcortical afferent connections of the squirrel monkey's (lateral) premotor cortex: evidence for visual cortical afferents. Int. J. Neurosci..

[bb0400] McIntyre C.C., Hahn P.J. (2010). Network perspectives on the mechanisms of deep brain stimulation. Neurobiol. Dis..

[bb0405] Miller K.L., Stagg C.J., Douaud G., Jbabdi S., Smith S.M., Behrens T.E.J., Jenkinson M., Chance S.A., Esiri M.M., Voets N.L., Jenkinson N., Aziz T.Z., Turner M.R., Johansen-Berg H., McNab J.A. (2011). Diffusion imaging of whole, post-mortem human brains on a clinical MRI scanner. NeuroImage.

[bb0410] Morel A., Magnin M., Jeanmonod D. (1997). Multiarchitectonic and stereotactic atlas of the human thalamus. J. Comp. Neurol..

[bb0415] Murata J.-I., Kitagawa M., Uesugi H., Saito H., Iwasaki Y., Kikuchi S., Tashiro K., Sawamura Y. (2003). Electrical stimulation of the posterior subthalamic area for the treatment of intractable proximal tremor. J. Neurosurg..

[bb0420] Nauta H.J. (1979). Projections of the pallidal complex: an autoradiographic study in the cat. NSC.

[bb0425] Ni Z., Pinto A.D., Lang A.E., Chen R. (2010). Involvement of the cerebellothalamocortical pathway in Parkinson disease. Ann. Neurol..

[bb0430] Nieuwenhuys R., Voogd J., van Huijzen C. (2013). The Human Central Nervous System.

[bb0435] Pahwa R., Lyons K.E., Wilkinson S.B., Tröster A.I., Overman J., Kieltyka J., Koller W.C. (2001). Comparison of thalamotomy to deep brain stimulation of the thalamus in essential tremor. Mov. Disord..

[bb0440] Parent A., De Bellefeuille L. (1982). Organization of efferent projections from the internal segment of globus pallidus in primate as revealed by fluorescence retrograde labeling method. Brain Res..

[bb0445] Percheron G., François C., Talbi B., Meder J.F., Fénelon G., Yelnik J. (1993). The primate motor thalamus analysed with reference to subcortical afferent territories. Stereotact. Funct. Neurosurg..

[bb0450] Petersen E.A., Holl E.M., Martinez-Torres I., Foltynie T., Limousin P., Hariz M.I., Zrinzo L. (2010). Minimizing brain shift in stereotactic functional neurosurgery. Neurosurgery.

[bb0455] Petersen M.V., Lund T.E., Sunde N., Frandsen J., Rosendal F., Juul N., Østergaard K. (2016). Probabilistic versus deterministic tractography for delineation of the cortico-subthalamic hyperdirect pathway in patients with Parkinson disease selected for deep brain stimulation. J. Neurosurg..

[bb0460] Plaha P., Khan S., Gill S.S. (2008). Bilateral stimulation of the caudal zona incerta nucleus for tremor control. J. Neurol. Neurosurg. Psychiatry.

[bb0465] Pollak P., Benabid A.L., Gervason C.L., Hoffmann D., Seigneuret E., Perret J. (1993). Long-term effects of chronic stimulation of the ventral intermediate thalamic nucleus in different types of tremor. Adv. Neurol..

[bb0470] Pouratian N., Zheng Z., Bari A.A., Behnke E., Elias W.J., Desalles A.A.F. (2011). Multi-institutional evaluation of deep brain stimulation targeting using probabilistic connectivity-based thalamic segmentation. J. Neurosurg..

[bb0475] Ramnani N., Behrens T.E.J., Penny W., Matthews P.M. (2004). New approaches for exploring anatomical and functional connectivity in the human brain. BPS.

[bb0480] Russchen F.T., Amaral D.G., Price J.L. (1987). The afferent input to the magnocellular division of the mediodorsal thalamic nucleus in the monkey, *Macaca fascicularis*. J. Comp. Neurol..

[bb0485] Sakai S.T., Inase M., Tanji J. (1999). Pallidal and cerebellar inputs to thalamocortical neurons projecting to the supplementary motor area in *Macaca fuscata*: a triple-labeling light microscopic study. Anat. Embryol..

[bb0490] Sakai S.T., Stepniewska I., Qi H.X., Kaas J.H. (2000). Pallidal and cerebellar afferents to pre-supplementary motor area thalamocortical neurons in the owl monkey: a multiple labeling study. J. Comp. Neurol..

[bb0495] Sammartino F., Krishna V., King N.K.K., Lozano A.M., Schwartz M.L., Huang Y., Hodaie M. (2016). Tractography-based ventral intermediate nucleus targeting: novel methodology and intraoperative validation. Mov. Disord..

[bb0500] Schaltenbrand G., Wahren W., Hassler R. (1977). Atlas for Stereotaxy of the Human Brain.

[bb0505] Schell G.R., Strick P.L. (1984). The origin of thalamic inputs to the arcuate premotor and supplementary motor areas. J. Neurosci..

[bb0510] Schuurman P.R., Bosch D.A., Merkus M.P., Speelman J.D. (2008). Long-term follow-up of thalamic stimulation versus thalamotomy for tremor suppression. Mov. Disord..

[bb0515] Sedrak M., Gorgulho A., Frew A., Behnke E., DeSalles A., Pouratian N. (2011). Diffusion tensor imaging and colored fractional anisotropy mapping of the ventralis intermedius nucleus of the thalamus. Neurosurgery.

[bb0520] Smith S.M. (2002). Fast robust automated brain extraction. Hum. Brain Mapp..

[bb0525] Smith S.M., Jenkinson M., Woolrich M.W., Beckmann C.F., Behrens T.E.J., Johansen-Berg H., Bannister P.R., De Luca M., Drobnjak I., Flitney D.E., Niazy R.K., Saunders J., Vickers J., Zhang Y., De Stefano N., Brady J.M., Matthews P.M. (2004). Advances in functional and structural MR image analysis and implementation as FSL. NeuroImage.

[bb0530] Sotiropoulos S.N., Jbabdi S., Xu J., Andersson J.L., Moeller S., Auerbach E.J., Glasser M.F., Hernandez M., Sapiro G., Jenkinson M., Feinberg D.A., Yacoub E., Lenglet C., Van Essen D.C., Ugurbil K., Behrens T.E.J., WU-Minn HCP Consortium (2013). Advances in diffusion MRI acquisition and processing in the Human Connectome Project. NeuroImage.

[bb0535] Spiegelmann R., Nissim O., Daniels D., Ocherashvilli A., Mardor Y. (2006). Stereotactic targeting of the ventrointermediate nucleus of the thalamus by direct visualization with high-field MRI. Stereotact. Funct. Neurosurg..

[bb0540] Stacy M.A., Elble R.J., Ondo W.G., Wu S.-C., Hulihan J., TRS Study Group (2007). Assessment of interrater and intrarater reliability of the Fahn-Tolosa-Marin Tremor Rating Scale in essential tremor. Mov. Disord..

[bb0545] Strick P.L. (1973). Light microscopic analysis of the cortical projection of the thalamic ventrolateral nucleus in the cat. Brain Res..

[bb0550] Tanaka D. (1976). Thalamic projections of the dorsomedial prefrontal cortex in the rhesus monkey (*Macaca mulatta*). Brain Res..

[bb0555] Tobias T.J. (1975). Afferents to prefrontal cortex from the thalamic mediodorsal nucleus in the rhesus monkey. Brain Res..

[bb0560] Traynor C.R., Barker G.J., Crum W.R., Williams S.C.R., Richardson M.P. (2011). Segmentation of the thalamus in MRI based on T1 and T2. NeuroImage.

[bb0565] Vassal F., Coste J., Derost P., Mendes V., Gabrillargues J., Nuti C., Durif F., Lemaire J.-J. (2012). Direct stereotactic targeting of the ventrointermediate nucleus of the thalamus based on anatomic 1.5-T MRI mapping with a white matter attenuated inversion recovery (WAIR) sequence. Brain Stimul..

[bb0570] Witjas T., Carron R., Krack P., Eusebio A., Vaugoyeau M., Hariz M., Azulay J.P., Régis J. (2015). A prospective single-blind study of Gamma Knife thalamotomy for tremor. Neurology.

[bb0575] Yarita H., Iino M., Tanabe T., Kogure S., Takagi S.F. (1980). A transthalamic olfactory pathway to orbitofrontal cortex in the monkey. J. Neurophysiol..

[bb0580] Yushkevich P.A., Piven J., Hazlett H.C., Smith R.G., Ho S., Gee J.C., Gerig G. (2006). User-guided 3D active contour segmentation of anatomical structures: significantly improved efficiency and reliability. NeuroImage.

