# Supplementary material for: Connectivity derived thalamic segmentation in deep brain stimulation for tremor
Source: Neuroimage Clin. 2018 Jan 28;18:130–42. doi: 10.1016/j.nicl.2018.01.008 (PMC5790021; doi:10.1016/j.nicl.2018.01.008)
Supplement: Supplementary Table 1 — Coordinates of template generated and patient specific dentate-thalamic clusters. [file mmc1.docx]

**Supplementary Table 1: Coordinates of template generated and patient specific dentate-thalamic clusters**

| **Patient** | **Side** | **Group dentate-thalamic cluster warped to patient space*** | | | | | | **Patient specific dentate-thalamic cluster in patient space*** | | | | | | **Variance between the two groups (mm)** | | | | | | **Euclidian distance between the two groups (mm)** | |
| --- | --- | --- | --- | --- | --- | --- | --- | --- | --- | --- | --- | --- | --- | --- | --- | --- | --- | --- | --- | --- | --- |
|  |  | Maximum intensity | | | Centre of gravity | | | Maximum intensity | | | Centre of gravity | | | Maximum intensity | | | Centre of gravity | | | Maximum intensity | Centre of gravity |
|  |  | **X** | **Y** | **Z** | **X** | **Y** | **Z** | **X** | **Y** | **Z** | **X** | **Y** | **Z** | **X** | **Y** | **Z** | **X** | **Y** | **Z** |  |  |
| **1** | **L** | 88.0 | 85.0 | 106.0 | 90.1 | 85.6 | 108.0 | 90.0 | 86.0 | 107.0 | 90.0 | 87.5 | 109.0 | 2.0 | 1.0 | 1.0 | 0.1 | 1.9 | 1.0 | 2.4 | 1.4 |
|  | **R** | 69.0 | 87.0 | 107.0 | 67.8 | 86.7 | 109.0 | 68.0 | 86.0 | 107.0 | 68.0 | 86.8 | 109.0 | 1.0 | 1.0 | 0.0 | 0.2 | 0.1 | 0.0 | 1.4 | 1.0 |
| **2** | **L** | 86.0 | 87.0 | 104.0 | 89.1 | 87.4 | 107.0 | 84.0 | 86.0 | 104.0 | 88.0 | 86.0 | 107.0 | 2.0 | 1.0 | 0.0 | 1.1 | 1.4 | 0.0 | 2.2 | 1.5 |
|  | **R** | 69.0 | 91.0 | 106.0 | 67.8 | 90.8 | 108.0 | 70.0 | 89.0 | 105.0 | 70.0 | 89.0 | 107.0 | 1.0 | 2.0 | 1.0 | 2.2 | 1.8 | 1.0 | 2.4 | 3.1 |
| **3** | **L** | 92.0 | 95.0 | 113.0 | 91.3 | 95.9 | 114.0 | 90.0 | 95.0 | 112.0 | 91.4 | 95.6 | 114.0 | 2.0 | 0.0 | 1.0 | 0.1 | 0.3 | 0.0 | 2.2 | 1.0 |
|  | **R** | 69.0 | 97.0 | 112.0 | 67.4 | 98.4 | 114.0 | 68.0 | 97.0 | 112.0 | 68.7 | 98.0 | 114.0 | 1.0 | 0.0 | 0.0 | 1.3 | 0.4 | 0.0 | 1.0 | 1.3 |
| **4** | **L** | 90.0 | 89.0 | 94.0 | 89.3 | 89.2 | 94.9 | 90.0 | 88.0 | 93.0 | 90.1 | 86.9 | 94.0 | 0.0 | 1.0 | 1.0 | 0.8 | 2.3 | 0.9 | 1.4 | 1.6 |
|  | **R** | 72.0 | 89.0 | 94.0 | 69.0 | 90.6 | 97.7 | 69.0 | 90.0 | 96.0 | 68.7 | 89.9 | 98.3 | 3.0 | 1.0 | 2.0 | 0.3 | 0.7 | 0.6 | 3.7 | 2.3 |
| **5** | **L** | 86.0 | 86.0 | 83.0 | 87.1 | 86.6 | 85.0 | 85.0 | 86.0 | 83.0 | 86.6 | 87.9 | 85.7 | 1.0 | 0.0 | 0.0 | 0.5 | 1.3 | 0.7 | 1.0 | 0.5 |
|  | **R** | 66.0 | 87.0 | 83.0 | 63.6 | 87.3 | 86.1 | 66.0 | 87.0 | 83.0 | 64.7 | 88.3 | 85.0 | 0.0 | 0.0 | 0.0 | 1.1 | 1.0 | 1.1 | 0.0 | 1.1 |
| **6** | **L** | 91.0 | 87.0 | 99.0 | 90.8 | 87.1 | 100.0 | 92.0 | 86.0 | 100.0 | 90.8 | 84.4 | 100.0 | 1.0 | 1.0 | 1.0 | 0.0 | 2.7 | 0.0 | 1.7 | 1.4 |
|  | **R** | 69.0 | 88.0 | 98.0 | 66.7 | 90.0 | 102.0 | 65.0 | 87.0 | 98.0 | 65.9 | 87.8 | 102.0 | 4.0 | 1.0 | 0.0 | 0.8 | 2.2 | 0.0 | 4.1 | 1.3 |
| **7** | **L** | 89.0 | 87.0 | 105.0 | 89.7 | 87.5 | 106.0 | 88.0 | 85.0 | 105.0 | 89.6 | 86.1 | 107.0 | 1.0 | 2.0 | 0.0 | 0.1 | 1.4 | 1.0 | 2.2 | 2.0 |
|  | **R** | 67.0 | 87.0 | 105.0 | 64.8 | 88.1 | 108.0 | 67.0 | 88.0 | 105.0 | 65.3 | 89.2 | 109.0 | 0.0 | 1.0 | 0.0 | 0.5 | 1.1 | 1.0 | 1.0 | 1.1 |
| **8** | **L** | 85.0 | 92.0 | 90.0 | 88.8 | 91.3 | 92.7 | 89.0 | 91.0 | 91.0 | 89.6 | 91.1 | 93.3 | 4.0 | 1.0 | 1.0 | 0.8 | 0.2 | 0.6 | 4.2 | 1.6 |
|  | **R** | 70.0 | 93.0 | 91.0 | 68.0 | 92.7 | 93.4 | 68.0 | 93.0 | 91.0 | 67.2 | 92.1 | 93.2 | 2.0 | 0.0 | 0.0 | 0.8 | 0.6 | 0.2 | 2.0 | 0.8 |
| **9** | **L** | 87.0 | 77.0 | 90.0 | 89.9 | 76.7 | 92.9 | 88.0 | 76.0 | 91.0 | 90.2 | 74.7 | 93.4 | 1.0 | 1.0 | 1.0 | 0.3 | 2.0 | 0.5 | 1.7 | 1.4 |
|  | **R** | 69.0 | 79.0 | 94.0 | 68.9 | 76.8 | 93.6 | 70.0 | 76.0 | 91.0 | 68.3 | 75.1 | 93.3 | 1.0 | 3.0 | 3.0 | 0.6 | 1.7 | 0.3 | 4.4 | 4.3 |
| **Average** |  |  |  |  |  |  |  |  |  |  |  |  |  | **1.5** | **0.9** | **0.7** | **0.6** | **1.3** | **0.5** | **2.2** | **1.6** |
| **STD** |  |  |  |  |  |  |  |  |  |  |  |  |  | **1.2** | **0.8** | **0.8** | **0.6** | **0.8** | **0.4** | **1.2** | **0.9** |

*(1mm isotropic voxels)
